# Pediatric Bell’s Palsy: Prognostic Factors, Management Strategy, and Treatment Outcomes

**DOI:** 10.3390/jcm14010079

**Published:** 2024-12-27

**Authors:** Lorenzo Di Sarno, Anya Caroselli, Benedetta Graglia, Francesco Andrea Causio, Antonio Gatto, Valeria Pansini, Natalia Camilla Di Vizio, Antonio Chiaretti

**Affiliations:** 1 Department of Pediatrics, Fondazione Policlinico Universitario A. Gemelli, IRCCS, 00168 Rome, Italy; lorenzodisarno1993@gmail.com (L.D.S.); pansini.valeria@gmail.com (V.P.); 2 Department of Pediatrics, Fondazione Policlinico Universitario A. Gemelli, IRCCS, Università Cattolica del Sacro Cuore, 00168 Rome, Italy; anya0196@gmail.com (A.C.); benedetta.graglia@gmail.com (B.G.); nataliacamilla.divizio01@icatt.it (N.C.D.V.); antonio.chiaretti@policlinicogemelli.it (A.C.); 3 Section of Hygiene, Department of Life Sciences and Public Health, Università Cattolica del Sacro Cuore, 00168 Rome, Italy; francescoandrea.causio@unicatt.it

**Keywords:** Bell’s palsy, children, facial nerve, House–Brackmann, steroids, prognosis

## Abstract

**Background/Objectives:** Bell’s palsy (BP) is a neurological disorder characterized by sudden unilateral peripheral facial paralysis. The etiology in children remains largely unknown, and standardized management strategies are lacking. The aim of this retrospective cohort study is to evaluate clinical features, laboratory markers, and therapeutic options associated with recovery to identify potential prognostic factors and validate therapeutic strategies, with a particular focus on the role of corticosteroids and vitamin supplementation. **Methods:** A retrospective cohort study was conducted on 88 children (aged < 18 years) diagnosed with BP at a single tertiary care center between 2010 and 2023. Clinical data, including House–Brackmann (HB) scores, were collected at baseline and at a two-month follow-up. Statistical analysis was performed to assess the associations between demographic, clinical, and laboratory parameters with recovery outcomes. Prednisone and vitamin supplementation were administered at the discretion of the treating pediatrician. **Results:** In total, 81.8% of patients achieved complete recovery at 2-month follow-up (HB grade 1). No significant associations were found between recovery and gender, age, side of paralysis, initial HB grade, or laboratory markers. However, the use of prednisone was associated with a higher rate of incomplete recovery (*p* = 0.024), with higher doses correlating with poorer outcomes (*p* = 0.022). Vitamin supplementation showed no significant impact. **Conclusions:** Our findings suggest that corticosteroid therapy may not be a disease-modifying factor that ultimately influences outcomes in pediatric BP. Further large-scale studies are needed to define evidence-based protocols for managing pediatric BP.

## 1. Introduction

Bell’s palsy (BP) is a neurological condition characterized by sudden unilateral peripheral facial palsy with no identifiable cause. Other specific recognizable causes of facial paralysis do not fit within this definition and include: traumatic, neoplastic, infectious, toxic, congenital, and iatrogenic etiologies [1].

It can affect individuals across all age groups, with an estimated annual incidence of 15–40 per 100,000 people [2], making it the most common cause of peripheral facial paralysis, responsible for 60–75% of cases [3]. In pediatric populations, however, the incidence is much lower, ranging from 5 to 21 per 100,000 children per year [4] and increasing proportionally with age [2]. The etiology of BP in the pediatric population remains largely unknown [5]. Some researchers have proposed an immune-mediated mechanism, possibly triggered by an infectious event such as reactivation of Herpes simplex virus type 1 [6], with consequent inflammation and edema of the facial nerve fibers [4]. Despite these suggestions, definitive evidence for a specific cause in children is lacking.

The diagnosis of Bell’s palsy in children is primarily a clinical one, focusing on the impairment of the seventh cranial nerve [7]. Manifestations may include altered facial expression, impairment of the anterior two-thirds of the tongue, hyperacusis, and dysfunction of the salivary and lacrimal glands [8]. However, additional investigations are often required, particularly in the emergency setting, where it is crucial to make a straight distinction between idiopathic etiology and secondary causes.

To rule out underlying causes and aid diagnosis, radiological imaging and laboratory tests may provide insightful items, although there is no standardized approach [3].

The prognosis of Bell’s palsy in pediatric patients is generally favorable, with most children improving within a few weeks and achieving spontaneous full recovery within 12 months [9]. In rare cases, children may have residual deficits that can affect their quality of life, including social interaction and speech development [5,10].

In adults, early corticosteroid therapy has long been considered the cornerstone of treatment, and prompt administration has been shown to improve outcomes [5]. Nonetheless, the efficacy of corticosteroids in pediatric patients remains controversial [11]. Various other treatment modalities have been proposed in the literature, including vitamin supplementation, which some authors have reported to be beneficial [12,13,14,15,16,17,18].

The lack of randomized controlled trials in children, combined with their generally high recovery rates even without any treatment, makes it difficult to give clear therapeutic recommendations [12]. As a result, there is still much disparity and variability in the management of pediatric BP.

Overall, pediatric BP presents a unique challenge due to its relatively low incidence, uncertain etiology, and the absence of standardized treatment protocols. The clinical prognosis is mostly excellent, but there is a lack of consensus in the scientific community regarding disease-modifying factors, including corticosteroids and vitamin supplementation.

The current gaps in the management and treatment strategies for Bell’s palsy in pediatric patients highlight the need for further research. In light of these unmet needs, the aim of this retrospective cohort study is to address these gaps in the literature by evaluating clinical features, laboratory markers, and therapeutic options associated with recovery in pediatric patients with BP in order to identify potential prognostic factors and validate therapeutic strategies. We had a special focus on the role of corticosteroids and vitamin supplementation, contributing to the development of evidence-based management strategies for pediatric BP.

## 2. Materials and Methods

### 2.1. Study Design and Participants

We conducted a retrospective cohort study evaluating the records of patients aged <18 years who presented with Bell’s palsy (BP) at the Pediatric Emergency Department (PED) of the Fondazione Policlinico A. Gemelli in Rome between January 2010 and December 2023.

Ethical approval for this study was obtained from the Ethics Committee of the Fondazione Policlinico Universitario Agostino Gemelli, Rome (ID 6520, approval date 27 June 2024). The parents of the studied patients were informed about the purpose of this study and signed an informed consent form for consenting access to children’s medical records and for processing personal data.

Patients with peripheral facial nerve palsy without an identifiable etiology were considered eligible. Demographic and clinical data were collected for all participants, including age, gender, affected side of the face, laboratory tests (complete blood count, serum biochemistry panel), and management details. Two months after the first assessment, a second outpatient clinical evaluation was performed in our institution, and clinical parameters were also collected as well.

The House–Brackmann (HB) facial nerve scoring system was used to assess the severity of facial paralysis at the initial PED evaluation and at 2-month outpatient follow-up. The HB scale ranges from 1 (normal facial function) to 6 (complete paralysis). The full HB scoring system is shown in Table 1.

At 2-month follow-up, the primary outcome was categorized as “complete” recovery (HB grade 1) or “incomplete” recovery (HB grade greater than 1).

At the discretion of the attending pediatrician, blood tests were performed in a subset of patients, when the initial clinical presentation was severe or when secondary causes of facial paralysis needed to be ruled out. When blood parameters were available, the neutrophil-to-lymphocyte ratio (NLR) and the platelet-to-lymphocyte ratio (TLR) were calculated. The specific laboratory markers we selected (NLR, TLR, and LDH) are widely described in the previous literature as potential prognostic factors in BP [3,19,20,21].

Pharmacological therapy was also administered at the discretion of the treating physician. In our institution, options included prednisone or vitamin supplementation.

Prednisone was prescribed in doses ranging from 1 mg/kg/day to 2 mg/kg/day. The treatment duration consisted of 7 days at full dosage, followed by a gradual tapering.

Regarding vitamin supplementation, a multivitamin supplement used in our institution was prescribed at the discretion of the treating physician. The age-related dosage of each vitamin is outlined as follows:

- Patients aged 1 to 3: B2 0.45 mg/day, B3 5.5 mg/day, B5 3 mg/day, B6 0.45 mg/day, B12 0.7 mcg/day, inositol 5 mg/day;
- Patients aged 4 to 6: B2 0.625 mg/day, B3 8.25 mg/day, B5 4.5 mg/day, B6 0.675 mg/day, B12 1.05 mcg/day, inositol 7.5 mg/day
- Patients aged 6 and above: B2 0.9 mg/day, B3 11 mg/day, B5 6 mg/day, B6 0.9 mg/day, B12 1.4 mcg/day, inositol 10 mg/day

The duration of vitamin supplementation was eight weeks.

### 2.2. Statistical Analysis

Categorical variables are reported as counts and percentages. Normality of distribution of continuous variables was tested by means of a Shapiro–Wilk test. Continuous variables are expressed as means and standard deviations or as median and interquartile ranges, if not normally distributed. Statistical comparisons between groups were made using the Chi-squared test, for categorical variables. Differences in normally distributed continuous variables were tested using the two-tailed unpaired Student’s *t* test, while the Wilcoxon rank-sum test or the Mann–Whitney U test was used for not normally distributed continuous variables, as appropriate.

A two-sided *p*-value < 0.05 was considered statistically significant. The statistical analysis was performed using STATA 18.0 software (Stata Corporation, College Station, TX, USA).

## 3. Results

**Figure 1:**
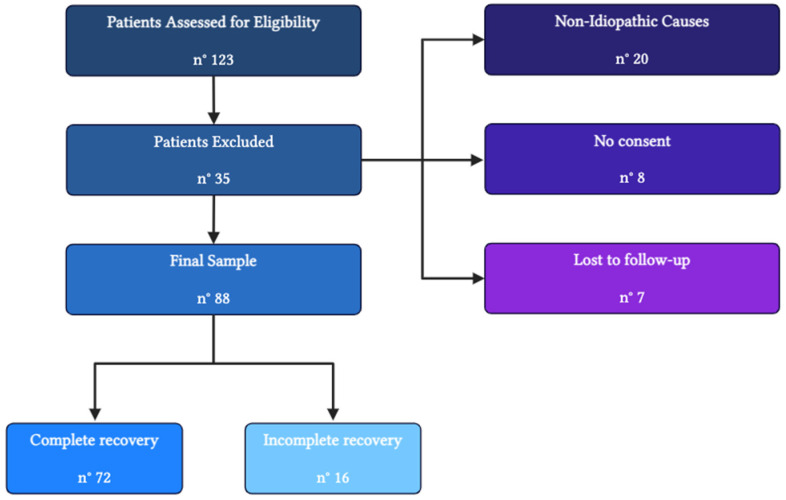
Study enrollment flowchart (created with biorender.com).

At the outset, a total of 123 children were eligible for inclusion in the study. However, 20 patients were excluded due to non-idiopathic causes of their facial paralysis (12 patients had post-traumatic forms, 5 had complicated mastoiditis, 1 had a neoplastic form, 1 had a demyelinating neuropathy, and 1 had Melkersson–Rosenthal syndrome), whereas 8 patients were excluded because their caregivers did not provide informed consent for enrollment. Furthermore, 7 patients were lost to follow-up during the study. The full enrollment flowchart is depicted in Figure 1.

Table 2 shows the epidemiological and clinical features of the study population, by total number and stratified by their final House–Brackmann (HB) score at 2-month follow-up. The final sample included 39 males (44.3%) and 49 females (55.7%).

Regarding the side of facial paralysis, 53.4% of patients had left-sided involvement, while 46.6% had right-sided involvement. The majority of children, 62 patients (70.5%), were prescribed corticosteroids, whereas 26 patients (29.5%) were not. Additionally, a smaller proportion of patients, 35.2%, received a vitamin supplementation. At 2-month follow-up, 72 out of 88 patients (81.8%) achieved complete recovery, as indicated by an HB score of 1. The remaining 16 patients (18.2%) had an HB score of 2, indicating incomplete recovery. Remarkably, none of the patients had a higher HB score at the follow-up assessment.

**Figure 2:**
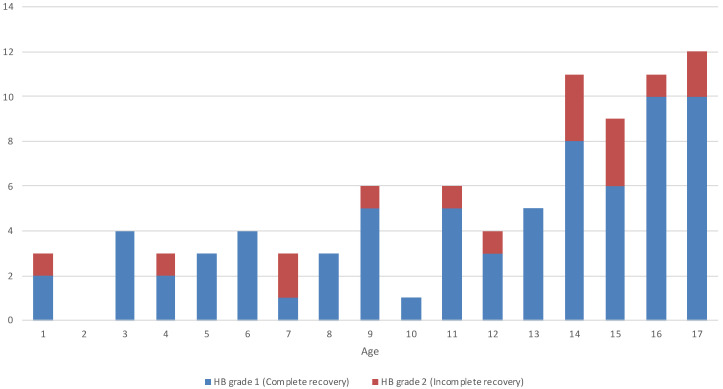
Age distribution of pediatric patients with Bell’s palsy, stratified by House–Brackmann (HB) grade at 2-month follow-up.

Gender was not significantly associated with HB grade at 2 months (Pearson chi-squared = 1.3533, *p* = 0.245). Age did not have a statistically significant association with HB grade at 2 months (Mann–Whitney U Test z-score = 0.69456, *p* = 0.4902, as shown in Table 3). Age distribution of pediatric patients with BP, stratified by HB grade at follow-up, is depicted in Figure 2.

No significant association was found between the side of paralysis (left or right) and the HB grade at 2 months (Pearson Chi-squared = 0.0634, *p* = 0.801). Moreover, the association between baseline HB grade and HB grade at 2 months was not statistically significant (Pearson Chi-squared = 1.9416, *p* = 0.747).

Vitamin supplementation did not have a significant relationship with HB grade at 2 months (Pearson Chi-squared = 0.1356, *p* = 0.713).

The relationship between prednisone use and HB grade at 2 months was statistically significant (Pearson Chi-squared = 5.0982, *p* = 0.024). Patients who received steroids had a higher rate of incomplete recovery (HB grade 2) compared to those who did not receive steroids (24.1% vs 3.8%), as shown in Table 4.

**Figure 3:**
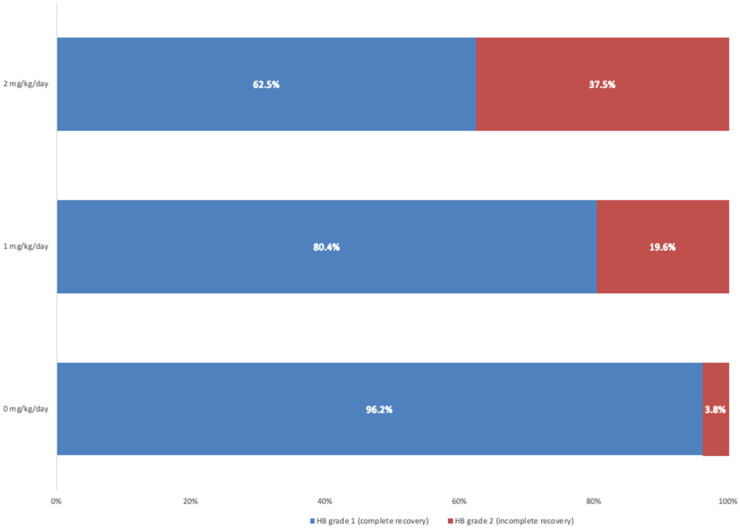
Percentage representation of the House–Brackmann (HB) score at two-month follow-up, stratified according to the prednisone dosage administered.

Furthermore, the Pearson Chi-squared test was applied to assess the relationship between prednisone dosage and HB grade outcomes as well (Table 5). We found a statistically significant association between prednisone dosage and recovery outcomes at two-month follow-up (Pearson Chi-squared = 7.88, *p* = 0.022). Specifically, patients who received a higher dose of steroids had higher rates of incomplete recovery (HB grade 2). The clinical outcome according to the HB score at two-month follow-up, stratified by prednisone dosage administered, is represented in Figure 3. The percentage of HB grade 2 increases proportionally with prednisone dose: 3.8% at 0 mg/kg/day, 19.6% at 1 mg/kg/day, and 37.5% at 2 mg/kg/day.

Three laboratory values were measured in a subset of patients (n = 24): neutrophil-to-lymphocyte ratio (NLR), thrombocyte-to-lymphocyte ratio (TLR), and lactate dehydrogenase (LDH). The complete results are shown in Table 6. The relationship between the laboratory values and the HB score at two months did not reveal statistically significant associations.

The Wilcoxon rank-sum test showed no significant difference in the neutrophil-to-lymphocyte ratio (NLR) between patients with HB grade 1 and HB grade 2 at 2 months (z = −0.268, *p* = 0.7884). Similarly, no significant difference was found in lactate dehydrogenase (LDH) levels between patients with HB grade 1 and HB grade 2 at 2 months using the Wilcoxon rank-sum test (z = −0.234, *p*= 0.8150). The thrombocyte-to-lymphocyte ratio (TLR) did not differ significantly between patients with HB grade 1 and HB grade 2 at 2 months using the Wilcoxon rank-sum test (z = 1.044, *p* = 0.2966).

## 4. Discussion

This is one of the largest retrospective studies to evaluate the factors associated with complete recovery in pediatric patients with BP.

Outcome determinants in adult patients with Bell’s palsy have been extensively studied, with complete resolution of the clinical picture in 80–94% of adults treated with or without antiviral therapy [4]. Although several prognostic factors have been identified in adults, the low incidence of Bell’s palsy in children has hindered effective research into clearly identifiable prognostic factors. In pediatrics, there is a paucity of evidence available both in terms of diagnosis and therapy [22]. Thus, standardized and unambiguous management through official guidelines is difficult to establish, similar to other pediatric conditions [23]. A comprehensive overview of the most recent studies on pediatric BP is shown in Table 7.

Previously, several studies in the literature have questioned the role of age, claiming that a younger patient recovers better than an older one under the same conditions [24,25]. Conversely, we have not confirmed this relationship. Other factors, i.e., baseline HB score or side of paralysis, were not significantly associated with the 2-month outcome. Similarly, Aysel et al. found no significant correlation between gender, affected side, initial HB score, and final recovery [3].

In terms of treatment, our study showed that the use of steroids was more associated with an incomplete recovery at 2-month follow-up. This statistical relationship ought to be interpreted as an association, rather than a causal inference, between steroid use and the HB grade at follow-up. However, these findings raise further questions about their disease-modifying role in this patient population. A bunch of previous studies in the literature have questioned the role of steroids in pediatric patients, arguing that their impact on outcome has not been clearly established [9,26,27,28]. In 2013, Ismail et al. conducted a retrospective longitudinal study with a sample size of 100 pediatric patients with facial palsy, 79 of whom had Bell’s palsy. There was no statistically significant difference in recovery time between the steroid-treated and untreated groups, pointing out the necessity to identify the specific circumstances in which steroids may be beneficial [26]. Conversely, Aysel et al., in 2020, recorded high recovery rates in children treated with corticosteroids, with 68.1% achieving complete recovery and 25.5% nearly complete recovery after six months of follow-up [3].

Ünüvar et al. performed a prospective randomized controlled study and did not find any statistically significant difference in outcome between pediatric patients with BP who received methylprednisolone and those who did not at a 4-month, 6-month, and 12-month follow-up [27].

In a first study, Babl et al. conducted a double-blind, placebo-controlled, randomized trial of prednisolone in children presenting with BP at the pediatric emergency department (PED). A vast number of patients had complete recovery of facial functions within six months, regardless of prednisolone use [28]. In a subsequent paper presenting follow-up data at 12 months, it was again shown that there was no statistically significant difference in the percentage of patients who experienced a complete recovery of facial function between the corticosteroid group and the placebo group (OR 3.12; 95% CI 0.61, 15.98) [9].

**Table 7:** A comprehensive overview of the most recent studies on pediatric Bell’s Palsy.

Author (Year)	Study Design	Population	Age	FU	Dose of Steroids	Outcome Measures	Recovery Rate-Steroids Received	Recovery Rate-Steroids NOT Received
Babl et al., 2024 [9]	RCT	187	6 months–18 years	6-mo 12-mo *	Oral PSL∼1 mg/kg/day up to 50 mg	CA HB grade MPA HB grade	CA 6-mo 98% MPA 6-mo 94% 12-mo 96%	CA 6-mo 93% MPA 6-mo 89% 12-mo 92%
Babl et al., 2022 [28]	RCT	187	6 months–18 years	1-mo 3-mo 6-mo	Oral PSL∼1 mg/kg/day up to 50 mg	HB grade	1-mo 49% 3-mo 90% 6-mo 98%	1-mo 57% 3-mo 85% 6-mo 93%
Yoo et al., 2021 [4]	RC	88	<19 years	6-mo	Oral PSL ∼1 mg/kg/day	HB grade	55.7%	N/A
Aysel et al., 2020 [3]	RC	47	7–17 years	6-mo	Oral or intravenous MP1 mg/kg/day	HB grade	complete recovery 68.1% complete + almost complete recovery 93.6%	N/A
Lee et al., 2020 [24]	RC	53	1–16.3 years	1-mo	Oral PSL or MP1 mg/kg/day	HB grade	complete recovery 56% complete + partialrecovery 96%	N/A
Karatoprak et al., 2019 [29]	RC	102	2–17 years	6-mo	oral PSL 1 vs. 2 mg/kg/day	HB grade	99%	N/A
Achour et al., 2015 [30]	RC	37	13.9 (mean)	N/M	Oral PSL 1 mg/kg/day	HB grade Freyss muscle testing	94.6%	N/A
Wang et al., 2010 [31]	RC	43	8.0 (mean)	1 year	Oral PDN <1 vs. 1 mg/kg/day	HB grade	96%	100%
Chen and Wong 2005 [25]	RC	32	0–15.5 years	N/M	Oral PSL 1–2 mg/kg/day	N/M	95.7%	100%
Unüvar et al., 1999 [27]	RCT	42	24 to 74-mo	4-mo 6-mo 12-mo	Oral MP 1 mg/kg/day	HB grade	4-mo 86% 6-mo 100% (12-mo improvement in all patients)	4-mo 72% 6-mo 86% (12-mo improvement in all patients)

CA: Clinician-administered; FU: follow-up; HB: House–Brackmann; mo: months; MP: Methylprednisolone; MPA: Modified parent-administer; N/A: Not applicable; N/M: Not Mentioned; PDN: prednisone; PSL: prednisolone; RCT: Randomized Control Trial; RC: Retrospective Cohort. * Only for modified parent-administered HB scale.

Our study showed a statistically significant association between the dose of prednisone administered and the HB score at 2 months. Focusing on this relationship, it is even clearer that the absence of therapy, defined as 0 mg/kg, is associated with a better outcome at two months compared to treated subjects who had remarkably lower resolution rates. Arican et al., in 2017, observed similar recovery rates between patients receiving different doses of steroids, reinforcing the notion that steroid therapy may not be a disease-modifying factor [32]. It is pivotal to emphasize that this relationship is correlational rather than causal. Our data indicate that patients receiving prednisone exhibited a higher rate of incomplete recovery, with statistically significant differences in clinical outcomes observed overall and even when dosage levels were split. However, these findings should not be misinterpreted as evidence that prednisone directly impairs recovery. It is essential to recognize that while associations can provide insights into potential trends, they do not establish definitive cause-and-effect relationships. We strongly recommend conducting future research through prospective, randomized studies to validate these findings and clarify the role of corticosteroids in managing pediatric Bell’s palsy. Such studies would provide a more robust framework for evaluating treatment efficacy and could lead to evidence-based protocols.

Our study also focused on the correlation between vitamin supplementation and HB scores at follow-up, but no significant association was found. However, it may be worthwhile to further investigate this relationship in larger sample sizes. Although vitamin supplementation is commonly used in the management of Bell’s palsy, it involves the most diverse combinations of B and non-B vitamins and is not regulated by guidelines or robust scientific evidence. The choice of vitamin type and duration of treatment is currently largely arbitrary [17].

There is limited evidence on the role of vitamins in recovery from Bell’s palsy, based on open trials, case controls, or case reports, but no randomized controlled trials.

In an open randomized trial by Jalaludin et al., 60 patients were divided into three treatment groups: steroid, methylcobalamin, and methylcobalamin + steroid [14]. The overall clinical presentation after 1–3 weeks of treatment was significantly more severe in the steroid group when compared to the methylcobalamin and methylcobalamin plus steroid groups.

According to a recent review by Hamayal et al., vitamin D deficiency is associated with a higher severity of BP and may, therefore, be a useful biomarker in assessing BP outcome [15]. Vitamin D plays a pivotal role in nerve repair and regeneration, including promoting nerve myelination by regulating genes, e.g., Prx and Tspan2 [16,17]. It also stimulates the production of neurotrophins, which are essential for neuronal survival and maintenance of neuronal function [18,33].

The overall high recovery rates typically observed in pediatric patients with Bell’s palsy may mask any subtle benefits that vitamins could confer. Furthermore, the potential individual variability in response to vitamin supplementation, even when dosages are standardized, may limit our ability to detect a significant effect across the population studied. Additionally, it is essential to consider that the biological mechanisms through which vitamins may influence nerve recovery are complex and not fully understood. Therefore, prospective studies with larger sample sizes and controlled variables would be beneficial to clarify these relationships and explore optimal dosages and timing for vitamin supplementation in pediatric Bell’s palsy patients. Regarding laboratory values, Aysel et al., in 2020, found relevance only for NLR, observing significantly higher rates of NLR in patients with a high baseline HB score [3]; however, no significant correlation was observed between TLR or LDH and the degree of facial paralysis.

A review and meta-analysis by Oya et al. in 2018, mainly in the adult population, focused on the relationship between NLR and TLR values and BP. NLR was found to be significantly higher in patients with BP compared to controls, and a higher NLR was observed in the non-recovery group, suggesting a possible association with poor prognosis and severity of facial nerve inflammation [19]. Conversely, this paper did not show any statistically significant correlations between TLR and the inflammatory state of BP, and most of the articles analyzed did not even assess the value of TLR in patients. Another meta-analysis by Kim et al. in 2021 further confirmed a significant relationship between elevated NLR values and adult patients with BP compared to controls [20]. In addition, a higher NLR value was found in the BP non-recovery group compared to the recovery group. TLR also showed a correlation with patients affected by BP, in comparison to the control group. However, no significant correlation was identified between TLR and severity, and no relationship with outcome was investigated. In one of the included studies [21], LDH was also found to be higher in the BP group compared to the control group, although no relationship with outcome was investigated.

The present study did not identify any statistically significant correlation between laboratory-derived values (NLR, TLR, LDH) and the HB scores at two-month follow-up. This finding may be partially due to the reduced subset of patients who underwent blood testing. Further investigation in larger samples is needed to gain insight into the potential associations between BP and these markers, as well as other potentially useful indicators [20].

Our study has limitations; first of all, its retrospective design. Retrospective studies are based on pre-existing medical records, which may introduce several potential biases. First, recall bias may affect the accuracy of the data, because patients’ symptoms, treatments, and outcomes were recorded based on past events, and the level of detail may vary between cases. Additionally, some data may be incomplete or missing from medical records. The variable quality and completeness of data in medical records could introduce missing information and inaccuracies, and limit the robustness of the analysis. In addition, retrospective studies often cannot identify the reasons for loss to follow-up, which can potentially bias results.

Second, another inherent limitation is the lack of randomization in treatment allocation: this limits our ability to establish causal relationships between treatment modalities and outcomes. As a matter of fact, our findings merely reflect associations rather than definitive evidence of efficacy or harm. A prospective study design, with standardized data collection protocols and randomized treatment allocation, would help mitigate these issues. Furthermore, there is wide heterogeneity in treatment approaches among different pediatricians, as corticosteroid and vitamin supplementation were administered at the discretion of the attending physician. This variation in clinical practice could impact the observed outcomes. Nevertheless, we used a standardized and widely used outcome measure, the HB grading system, to assess the severity of facial paralysis and recovery outcomes. This shared tool ensures consistency and comparability across patients and studies.

Third, our study had a relatively small sample size, which limits the generalizability of the findings and the statistical power to detect smaller effects or associations. Nevertheless, to the best of our knowledge, our study includes a relatively large sample size (n = 88) compared to other studies focusing only on pediatric BP [24,25,30,31,34,35]. Given the lower incidence of Bell’s palsy in children, assembling such a cohort is challenging, which enhances the relevance of our findings in the pediatric population. A major limitation is the small subset of patients who underwent laboratory testing, which was primarily performed at the discretion of the treating physician. This selection may introduce bias, as the laboratory data may disproportionately represent patients with more severe or atypical clinical presentations.

Fourth, our study was conducted in a single center, which does not allow generalization of our findings to the entire pediatric population and limits the types of approaches and protocols analyzed to those used in our institution.

Lastly, the relatively brief timeframe for follow-up may have underestimated some patients who experienced a more delayed recovery. For this reason, the original goal was to follow up with patients at 6 months. However, a significant number of patients were lost to follow-up early, which led us to adopt this shorter cut-off, similar to other pediatric studies in the literature [24,34].

## 5. Conclusions

This study investigated potential prognostic factors and treatment outcomes in pediatric Bell’s palsy. Our findings suggest that the use of corticosteroids may not significantly improve recovery outcomes.

No significant correlations were observed between gender, age, side of facial paralysis, initial House–Brackmann grade, use of vitamins or supplements, laboratory markers, and recovery outcome. Given the lack of standardized guidelines and limited pediatric-specific research, further large-scale studies are needed to establish evidence-based management protocols for pediatric Bell’s palsy.

## Figures and Tables

**Table 1:** House–Brackmann grading of facial nerve palsy.

Grade	Clinical Features
**I**	Normal: Normal function in all areas.
**II**	Mild Dysfunction: Slight weakness noticeable on close inspection; may have slight synkinesis. Normal symmetry and tone at rest. -Forehead: Moderate to good function. -Eye: Complete closure with minimal effort. -Mouth: Slight asymmetry.
**III**	Moderate Dysfunction: Obvious but not disfiguring differences between the two sides; noticeable but not severe synkinesis, contracture, or hemifacial spasm. Normal symmetry and tone at rest. -Forehead: Slight to moderate movement. -Eye: Complete closure with effort. -Mouth: Slightly weak with maximum effort.
**IV**	Moderately Severe Dysfunction: Obvious weakness and/or disfiguring asymmetry; normal symmetry and tone at rest. -Forehead: None. -Eye: Incomplete closure. -Mouth: Asymmetric with maximum effort.
**V**	Severe Dysfunction: Only barely perceptible motion; asymmetry at rest. -Forehead: None. -Eye: Incomplete closure. -Mouth: Slight movement.
**VI**	Total Paralysis: No movement.

**Table 2:** Demographic and clinical characteristics of pediatric patients with Bell’s palsy, stratified by House–Brackmann (HB) grade at 2-month follow-up (FU).

	Total (n = 88)	HB Grade 1 at 2 Month FU (n = 72)	HB Grade 2 at 2 Month FU (n = 16)	*p*-Value
Gender, n (%)				
Male	39 (44.3)	34 (47.2)	5 (31.2)	0.245
Female	49 (55.7)	38 (52.8)	11 (68.8)	
Side, n (%)				
Left	47 (53.4)	38 (52.8)	9 (56.3)	0.801
Right	41 (46.6)	34 (47.2)	7 (43.7)	
Steroids, n (%)				
No	26 (29.5)	25 (34.7)	1 (6.3)	0.024
Yes	62 (70.5)	47 (65.3)	15 (93.7)	
Vitamin supplementation, n (%)				
No	57 (64.8)	46 (63.9)	11 (68.8)	0.713
Yes	31 (35.2)	26 (36.1)	5 (31.2)	
HB at baseline, n (%)				
1	1 (1.1)	1 (1.4)	0 (0.0)	0.747
2	13 (14.8)	12 (16.7)	1 (6.2)	
3	39 (44.3)	32 (44.4)	7 (43.8)	
4	32 (36.4)	25 (34.7)	7 (43.8)	
5	3 (3.4)	2 (2.8)	1 (6.2)	

**Table 3:** Age distribution of pediatric patients with Bell’s palsy, stratified by House–Brackmann (HB) grade at 2-month follow-up (FU).

Age, n (%)	HB Grade 1 at 2 Month FU	HB Grade 2 at 2 Month FU	Total
1	2 (2.78)	1 (6.25)	3 (3.41)
3	4 (5.56)	0 (0.0)	4 (4.55)
4	2 (2.78)	1 (6.25)	3 (3.41)
5	3 (4.17)	0 (0.0)	3 (3.41)
6	4 (5.56)	0 (0.0)	4 (4.55)
7	1 (1.39)	2 (12.5)	3 (3.41)
8	3 (4.17)	0 (0.0)	3 (3.41)
9	5 (6.94)	1 (6.25)	6 (6.82)
10	1 (1.39)	0 (0.0)	1 (1.14)
11	5 (6.94)	1 (6.25)	6 (6.82)
12	3 (4.17)	1 (6.25)	4 (4.55)
13	5 (6.94)	0 (0.0)	5 (5.68)
14	8 (11.1)	3 (18.75)	11 (12.5)
15	6 (8.33)	3 (18.75)	9 (10.23)
16	10 (13.89)	1 (6.25)	11 (12.5)
17	10 (13.89)	2 (12.5)	12 (13.64)
Total	72 (81.82)	18 (18.18)	88 (100)
Age ^a^	13 (8–16)	14 (8.5–15)	
Mann–Whitney U Test, z-score = 0.69456, *p* = 0.490			

^a^ Data are expressed as medians and interquartile ranges.

**Table 4:** Relationship between prednisone therapy and House–Brackmann (HB) grade at 2-month follow-up (FU) in pediatric patients with Bell’s palsy.

Prednisone Therapy	HB Grade 1 at 2 Month FU	HB Grade 2 at 2 Month FU	Total
No	25	1	26
Yes	47	15	62
Total	72	16	88
Pearson chi-squared = 5.09, *p* = 0.024			

**Table 5:** Prednisone dosage distribution of pediatric patients with Bell’s palsy, stratified by House–Brackmann (HB) grade at the 2-month follow-up (FU).

Administered Prednisone Dose	HB Grade 1 at 2 Month FU	HB Grade 2 at 2 Month FU	Total
0 mg/kg/day	25	1	26
1 mg/kg/day	37	9	46
2 mg/kg/day	10	6	16
Total	72	16	88
Pearson chi-squared = 7.88, *p* = 0.022			

**Table 6:** Descriptive statistics for laboratory values: neutrophil-to-lymphocyte ratio (NLR), thrombocyte-to-lymphocyte ratio (TLR), and lactate dehydrogenase (LDH) in pediatric patients with Bell’s palsy, stratified by House–Brackmann (HB) grade at 2-month follow-up (FU).

	Observations	HB Grade 1 at 2 Month FU ^a^	HB Grade 2 at 2 Month FU ^a^	Wilcoxon Statistic (z)	*p*-Value
NLR	24	1.26 (0.79–2.27)	1.36 (1.07–2.01)	−0.268	0.788
TLR	24	101.41 (75.65–120.32)	79.56 (71.70–98.62)	−0.234	0.815
LDH	22	185.00 (152.00–241.00)	225.00 (135.00–246.00)	1.044	0.297

^a^ Data are expressed as medians and interquartile ranges.

## Data Availability

The datasets presented in this article are not readily available because the data are part of an ongoing study. Requests to access the datasets should be directed to the corresponding author.
